# Elevated Gene Expression of Interleukin-32 Isoforms Alpha, Beta, Gamma, and Delta in the Peripheral Blood of Chronic Psoriatic Patients

**DOI:** 10.3390/diseases6010021

**Published:** 2018-03-14

**Authors:** Hani A. Al-Shobaili, Zafar Rasheed

**Affiliations:** 1 Department of Dermatology, College of Medicine, Qassim University, P.O. Box 6655, Buraidah 51452, Saudi Arabia; hani@qumed.edu.sa; 2 Department of Medical Biochemistry, College of Medicine, Qassim University, P.O. Box 6655, Buraidah 51452, Saudi Arabia

**Keywords:** IL-32, IL-32 isoforms, psoriasis, peripheral blood, inflammation

## Abstract

Inflammatory-mediated reactions have been implicated as contributors in a number of dermatological disorders, including psoriasis. However, the potential of interleukin (IL)-32 and its isoforms to contribute to the pathogenesis of psoriasis remains unexplored. This study was undertaken to investigate the role of IL-32 and its isoforms IL-32α, IL-32β, IL-32γ, and IL-32δ in the peripheral blood of psoriatic patients. The majority of chronic plaque psoriatic patients showed elevated IL-32 mRNA levels in the peripheral blood mononuclear cells (PBMCs) as compared with the levels of IL-32 mRNA in PBMCs of healthy controls (*p* = 0.001). To further investigate the role of elevated levels of IL-32 in psoriatic patients, IL-32 isoforms mRNAs were determined. All tested isoforms IL-32α, IL-32β, IL-32γ, and IL-32δ were overexpressed in psoriatic patients PBMCs as compared with healthy controls’ PBMCs (*p* < 0.05). IL-32α mRNA expression was also significantly higher as compared with all other isoforms of IL-32 in PBMCs of psoriatic patients (*p* < 0.001). In short, this is the first study that shows the role of IL-32 and its isoforms in the peripheral blood of psoriatic patients. Our novel findings support an association between elevated levels of IL-32 and psoriasis. The data also suggest that a major proinflammatory response of IL-32 may derive from IL-32α isoform in psoriasis.

## 1. Introduction

Psoriasis is a chronic disorder of the skin that often affects the joints. Onset of psoriasis has a significant impact on a patient’s quality of life and can lead to depression [1,2]. Inflammation plays a key role in its onset and it is now considered a multi-organ disorder with a well-characterized pathology [1,2]. Recent reports suggested that 2–5% of the world’s population has been affected by psoriasis; it has multiple types including extra-cutaneous manifestations that further support the involvement of multiple factors [1,2,3]. Psoriasis has been classified on the basis of clinical phenotypes, disease morphology, disease severity, and patient age [2]. Psoriasis vulgaris or plaque psoriasis is the most common form of psoriasis and covers almost 90% of all psoriatic patients [3]. In these patients, lesions generally start as erythematous macules or papules, extend peripherally, and coalesce to form plaques. The lesions may have different shapes/patterns of presentation that may appear anywhere on the body but have been commonly distributed in a symmetrical pattern especially on elbows, knees, and scalp [4].

The role of pro-inflammatory cytokines and other inflammatory mediators has been well reported in promoting cutaneous inflammation in patients with psoriasis [1,5]. Tumor necrosis factor (TNF)-α is well known to upregulate the expression of proinflammatory mediators, adhesion molecules, lymphocyte trafficking, and epidermal hyperplasia, which further promotes inflammation in psoriatic lesions [5]. Importantly, anti-TNF-α therapy was found to be an effective treatment for psoriasis [5,6], which provided proof for the involvement of proinflammatory mediators in promoting cutaneous inflammation in these patients. Furthermore, upregulation of interleukin (IL)-15 has also been documented in psoriatic skin and its blockade caused a marked reduction in psoriatic features [7]. Moreover, dysfunctions of various other cytokines such as IL-1β, IL-6, IL-17, IL-22, interferon (IFN)-γ, chemokine IL-8, and CXCL2 have also been documented in the psoriatic skin of these patients [8,9,10], which further supports the involvement of inflammation in psoriasis.

Interleukin-32 is a comparatively newly discovered cytokine and studies on it are still in their early stages. Studies have shown it is produced by various cell types including T lymphocytes, natural killer cells, monocytes, and epithelial cells [11,12,13,14,15]. Of particular importance, IL-32 exhibits numerous classical proinflammatory activities [13,14,15,16,17,18]. It stimulates the production of numerous proinflammatory cytokines including TNF-α, IL-1β, and IL-6 [13,19,20,21,22]. It also activates key proinflammatory cell signaling pathways such as mitogen-activated protein kinase (MAPK)-p38, extracellular receptor kinases (ERK), and nuclear transcription factor (NF)-κB in various cell types [22]. Kim et al. determined through a blast program of NCBI gene data bank that IL-32 is present in human chromosome 16p13.3 and has four isoform variants, IL-32α, IL-32β, IL-32γ, and IL-32δ [13]. Later on it was discovered that IL-32 gene has nine isoforms variants [16]. Among all IL-32 isoforms, IL-32α is the most abundant cDNA clone and it is reported that major proinflammatory activity of IL-32 has been derived from IL-32α as IL-32α is involved in the activation of p38-MAPK, EKR1/2, and JAK/STAT3 signaling, and also in the nuclear translocation of active subunits of NF-κB [13,23,24].

The proinflammatory role of IL-32 has been well reported in several disorders including rheumatoid arthritis, cancers, pulmonary tuberculosis, etc. [21,22,23,24,25,26,27], and also in many skin disorders such as atopic dermatitis, hidradenitis suppurativa, leishmaniasis, and systematic lupus erythematosus [28,29,30]. Most importantly, elevated IL-32 has also been reported in skin biopsies of psoriasis patients [31]. However, the role of IL-32 in the peripheral blood of psoriatic patients has never been investigated. In this study, we have addressed for the first time the possible role of IL-32 and its isoforms IL-32α, IL-32β, IL-32γ, and IL-32δ in the peripheral blood of psoriatic patients. Our findings indicate that IL-32 and all its isoforms may be pro-inflammatory mediators in psoriatic patients. 

## 2. Material and Methods

### 2.1. Patient Recruitment

The study was carried out in accordance with the Code of Ethics of the World Medical Association (Declaration of Helsinki as revised in Tokyo 2004) for humans and was approved by The General Directorate of Health Affairs, Al-Qassim Region, Ministry of Health, KSA (Ethical Approval # 45/44/781515; Registration # H-04-Q-001). Study subjects were recruited through the dermatology outpatient clinics of Qassim University. Patient selection was based on a clinical diagnosis of chronic plaque psoriasis, as described previously [32]. Only patients with uncomplicated chronic plaque psoriasis were included. Patients with pre-existing coronary artery disease, liver disease, renal disease, diabetes mellitus, hypertension, rheumatoid arthritis, and receiving systemic treatment for psoriasis (acitretin, ciclosporin, methotrexate, phototherapy, or biologics) for at least six weeks were excluded from the study. The inclusion criteria for controls were the absence of any prior history of psoriasis lesions. Informed consent from all selected patients was taken before sample collection. Venous blood samples from psoriatic patients (*n* = 42; age 35.3 ± 14.4 years) and healthy human subjects (*n* = 38; age 29.3 ± 9.4 years) were collected and the desired blood components were isolated on the same day of blood withdrawal for patients or controls.

### 2.2. Preparation of PBMCs, Reverse Transcription, and Real-Time Quantitative PCR

Blood samples from chronic psoriatic patients and from healthy human controls were collected in EDTA-treated vials and PBMCs were isolated by standard Ficoll density-gradient centrifugation using histopaque-1077 reagent (catalog # 10771; Sigma-Aldrich, St. Louis, MO, USA), as described previously [33]. Briefly, the histopaque (~5 mL) in a 15-mL centrifuge tube and gently layer the collected blood (~5 mL) on top of the histopaque using a 1 mL auto pipette. The layering must be performed gently so that the blood and histopaque form two separate layers. Centrifuge the tubes at 100× *g* for 30 min under refrigerating conditions. Immediately aspirate the whitish buffy coat containing PBMCs (~1.2 mL), which is formed by the interphase of the histopaque and medium. Wash the isolated PBMCs by centrifugation twice by sterile phosphate buffer saline (PBS, pH 7.4). Total RNA was prepared from isolated PBMCs by mirVana RNA isolation kit (catalog # AM1560; Ambion, Foster City, CA, USA). Moreover, RNA samples were further treated with DNA-free digested RNA column as per the manufacturer’s instructions (Ambion). Furthermore, RNA quality was monitored at 260 nm and 280 nm using a Thermo Scientific NanoDrop Spectrophotometer (Waltham, MA, USA) and only those RNA samples were processed that showed absorbance ratio 260/280 nm of ~2.0. Single-stranded cDNA was synthesized using the genomic DNA-free total RNA, prepared using 1.0 μg total RNA and the High-Capacity cDNA Reverse Transcription Kit using SuperScript First Strand cDNA synthesis kit (Applied Biosystems, Foster City, CA, USA), as described previously [34,35]. PCR amplification was carried out by TaqMan assay (Applied Biosystems, Foster City, CA, USA) using Step One Real-Time PCR System (Applied Biosystems). Typical profile times used were: initial step, 95 °C for 10 min, followed by a second step at 95 °C for 15 s and 60 °C for 60 s for 40 cycles with melting curve analysis. Expression levels were determined in one plate for each set of experiments. Values were normalized to the corresponding GAPDH or β-actin expression level measured within the same plate. Relative expression levels were analyzed using the ∆∆CT method, as described previously [36]. Primers used for PCR amplification have been summarized in Table 1.

### 2.3. Statistical Analysis

Statistical comparisons were performed by t two-tailed test using Mann–Whitney analysis or one-way ANOVA analysis followed by Tukey’s post hoc analysis using Graph Pad Prism-5 (San Diego, CA, USA); *p* < 0.05 was considered significant. Values written are mean ± SEM unless stated otherwise.

## 3. Results

### 3.1. Gene Expression of IL-32 in PBMCs of Chronic Psoriatic Patients 

**Figure 1:**
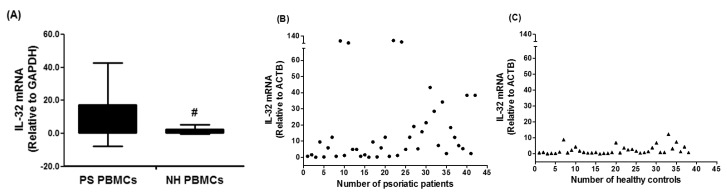
Gene expression of human interleukin (IL)-32 in the peripheral blood mononuclear cells (PBMCs) of chronic psoriatic patients. Data in column chart (**A**) and scattered data points chart (**B**,**C**). Expression of IL-32 mRNA was determined by real-time qRT-PCR using either GAPDH or ACTB as an endogenous control in the comparative ∆∆CT method. PS PBMCs, psoriatic patients PBMCs. NH PBMCs, normal human PBMCs; Data are representative (mean ± SD) of experiments with PBMCs obtained from psoriatic patients (*n* = 42) and normal healthy subjects (*n* = 38). # *p* = 0.001 versus PS PBMCs. Comparison analysis was performed using a two-tailed t-test using Mann–Whitney analysis.

**Figure 2:**
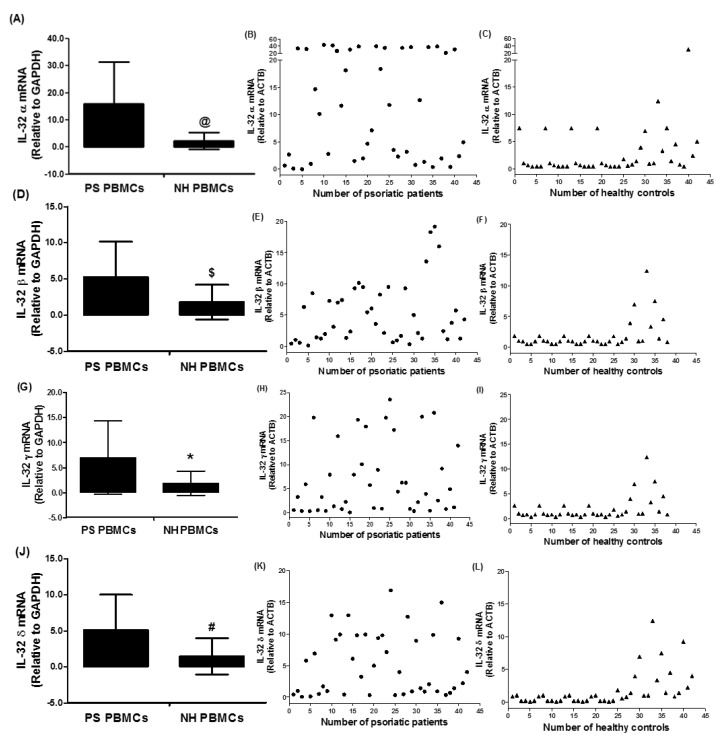
Gene expression of human interleukin (IL)-32 subtypes in the peripheral blood mononuclear cells (PBMCs) of chronic psoriatic patients. (**A**–**C**) Gene expression of IL-32 alpha (IL-32 α) in PS PBMCs and NH PBMCs. ^@^ *p* < 0.01 versus PS PBMCs. (**D**–**F**) Gene expression of IL-32 beta (IL-32 β) in PS PBMCs and NH PBMCs. ^$^ *p* < 0.05 versus PS PBMCs. (**G**–**I**) Gene expression of IL-32 gamma (IL-32 γ) in PS PBMCs and NH PBMCs. * p < 0.05 versus PS PBMCs. (**J**–**L**) Gene expression of IL-32 delta (IL-32 δ) in PS PBMCs and NH PBMCs. ^#^ *p* < 0.05 versus PS PBMCs. Expression of IL-32 subtypes was determined by real-time qRT-PCR using either GAPDH or β-actin as an endogenous control in the comparative ∆∆CT method. Data in column chart are representative (mean ± SD) of experiments with PBMCs obtained from psoriatic patients (*n* = 42) and normal healthy subjects (*n* = 38). PS PBMCs, psoriatic patients PBMCs; NH PBMCs, normal human PBMCs. Comparison analysis was performed using a two-tailed t-test using Mann–Whitney analysis.

In an attempt to understand the role of IL-32 in the pathogenesis of psoriasis, this is the first study to determine the role of IL-32 in the peripheral blood of psoriatic patients. Forty two patients with chronic plaque psoriasis were selected and IL-32 mRNA levels were determined by TaqMan assay using real-time PCR. The majority of psoriatic patients showed elevated mRNA level of IL-32 as compared to the IL-32 mRNA in PBMCs of normal healthy subjects. The average relative quantitation (±) SD of IL-32 mRNA levels relative to GAPDH in PBMCs from psoriatic patients and normal healthy subjects were 17.47 ± 25.30 and 2.49 ± 3.01 RQ, respectively (Figure 1A). Results are also presented as scattered data points (Figure 2B). The data reveal striking differences of IL-32 mRNA in the peripheral blood of psoriasis patients and healthy humans (*p* = 0.001).

### 3.2. Expression of IL-32 Isoforms α, β, γ, and δ in PBMCs of Chronic Psoriatic Patients 

To further investigate the role of elevated levels of IL-32 in psoriatic patients, IL-32 isoforms mRNA levels were determined. Results reveal that tested IL-32 isoforms, IL-32α, IL-32β, IL-32γ, and IL-32δ were expressed in the PBMCs of psoriatic patients as well as in those of healthy humans (Figure 2). The average relative quantitation (±) SD of IL-32α mRNA level in PBMCs from psoriatic patients and normal healthy subjects was 15.87 ± 15.52 and 2.06 ± 2.71 RQ, respectively (Figure 2A,B). The average relative quantitation (±) SD of IL-32β mRNA level in PBMCs from psoriatic patients and normal healthy subjects was 5.25 ± 4.92 and 1.83 ± 2.40 RQ, respectively (Figure 2C,D), whereas the IL-32γ mRNA level in PBMCs from psoriatic patients and normal healthy subjects was 7.00 ± 7.34 and 1.89 ± 2.42 RQ, respectively (Figure 2E,F). The average relative quantitation (±) SD of IL-32δ mRNA level in PBMCs from psoriatic patients and normal healthy subjects was 5.12 ± 4.90 and 1.49 ± 2.53 RQ, respectively (Figure 2G,H). These data pointed out that all isoforms of IL-32 α, β, γ, and δ were significantly overexpressed in the PBMCs of psoriatic patients as compared with PBMCs from healthy human controls (Figure 2; *p* < 0.05).

### 3.3. Overexpression of IL-32α in Psoriatic Patients

**Figure 3:**
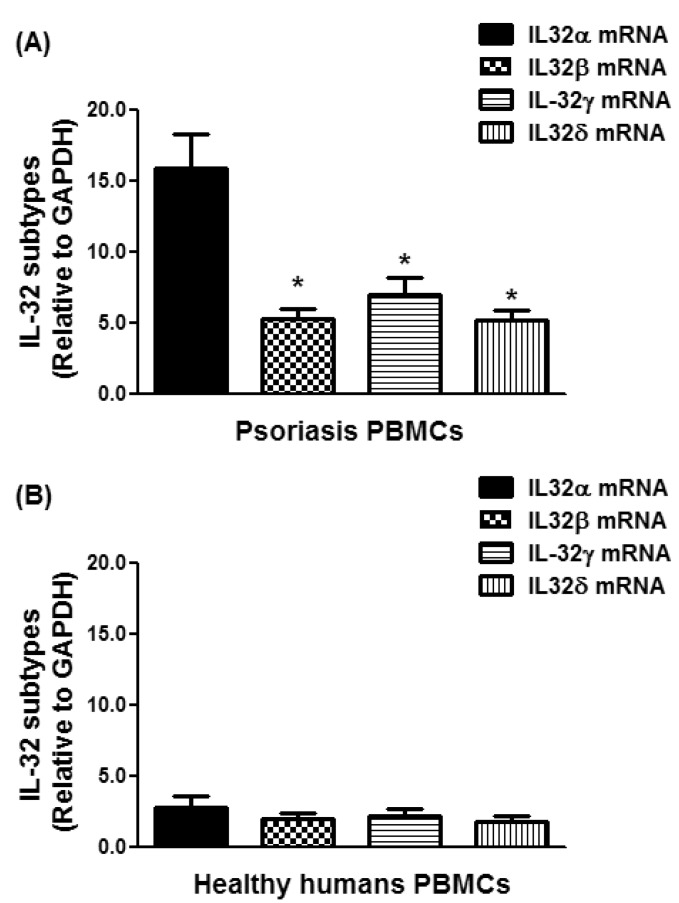
Overexpression of IL-32α mRNA in the peripheral blood mononuclear cells (PBMCs) of chronic psoriasis patients. (**A**) Gene expression of IL-32 isoforms in PBMCs from psoriasis patients. * *p* < 0.001 versus IL-32α mRNA; (**B**) gene expression of IL-32 isoforms in PBMCs from normal human subjects. The mRNAs of IL-32 isoforms were determined by real-time qRT-PCR using either GAPDH or β-actin as an endogenous control in comparative ∆∆CT method. Results are representative (mean ± SEM) and statistical analysis was performed using one-way ANOVA followed by Tukey’s multiple comparison tests.

In an attempt to determine the most active isoform form of IL-32 in the peripheral blood of psoriatic patients, the gene expression levels of all IL-32 isoforms were compared. The average relative quantitation (±) SD of mRNA of IL-32α, IL-32β, IL-32γ, and IL-32δ in PBMCs from psoriatic patients was 15.87 ± 15.52, 5.25 ± 4.92, 7.00 ± 7.34, and 5.12 ± 4.90 RQ, respectively (Figure 3A), whereas that of mRNA of IL-32α, IL-32β, IL-32γ, and IL-32δ in PBMCs of healthy human individuals was 2.06 ± 2.72, 1.83 ± 2.34, 1.89 ± 2.42, 1.49 ± 2.53 RQ, respectively (Figure 3B). Data pointed out that IL-32α mRNA expression was significantly higher compared with all other isoforms of IL-32 in PBMCs of psoriatic patients (Figure 3A; *p* < 0.001). In healthy PBMCs, all IL-32 isoforms were expressed almost equally (Figure 3B; *p* > 0.05). These results support the potential role of IL-32α in the pathogenesis of psoriasis.

## 4. Discussion

This is, to the best of our knowledge, the first study that demonstrated the role of IL-32 and its isoforms IL-32α, IL-32β, IL-32γ, and IL-32δ in the peripheral blood of psoriatic patients. The role of inflammation and cytokines in psoriasis is well defined; however, only a few, cytokines particularly TNF-α, have reached significant levels in clinical trials [5,6,8,9,10]. IL-15 is also overexpressed in psoriatic lesions compared with normal or pre-psoriatic skin [7]. Moreover, the blockade of IL-15 in psoriatic animal model attenuated the severity of the disease [7]. The pathogenic role of IL-18, IL-12, and IL-23 has been reported in psoriatic lesions, as treatment of psoriatic patients with UVB therapy reduces their levels in psoriatic lesions [37]. Furthermore, local administration of IL-10 reverses the pathological signatures of psoriasis [38]. Abnormal activities of IL-20 subfamily of cytokines have also been described in psoriasis [39]. IL-19, IL-20, IL-22, and IL-24 belong to the IL-20 subfamily of cytokines and their overexpression in psoriatic lesions has already been correlated with psoriatic pathology [39,40]. Moreover, leukocytes and epithetical cells treated with IL-20 subfamily members upregulate the production of other cytokines and various other proinflammatory mediators [39,40]. Furthermore, monocytes treated with IL-19 upregulate the production of TNF-α and IL-6 [41]. Elevated expression of IL-20 in transgenic mice produced skin abnormalities similar to psoriasis [42]. Moreover, a potential role of IL-22 has also been well defined in the onset of psoriatic lesions, as it induces the production of a number of proinflammatory mediators [43]. In the last decade, many other cytokines such as INF-γ, IL-17, etc. and chemokines such as IL-8, CXCL11, CCL5, etc. have also been reported in psoriatic pathogenesis [8,9,10]. Despite these important implications of cytokines and chemokine in psoriasis and the availability of advanced molecular approaches, the etiology of the psoriasis remains to be completely defined.

In this study the role of cytokine IL-32 was demonstrated in psoriatic patients. IL-32 was first discovered in IL-2-activated T cells and natural killer cells [11,12,13] and now it is well established that IL-32 exhibits numerous classical proinflammatory activities [11,12,13,14,15,16,17,18,19,20,21,22]. It stimulates the production of TNF-α, IL-1β, IL-6, INF-γ, IL-8, etc. [11,12,13]. Elevated levels of IL-32 have also been detected in endothelial cells from various origins [11]. Overexpression of IL-32 has also reported in fibroblast-like kidney cells COS-7 [11]. In recent decades, abnormalities in the expression and production of IL-32 have been identified in many human disorders such as rheumatoid arthritis, various cancers, pulmonary tuberculosis, atopic dermatitis, leishmaniasis, etc. [21,22,23,24,25,26,27,28,29,30]. Importantly, an elevated IL-32 level was found in the sera of patients with atopic dermatitis, asthmatic patients, systemic lupus erythematosus, etc. [27,28,30], indicating that IL-32 was produced by blood cells. However, the role of IL-32 in the peripheral blood has never been investigated in psoriasis. In an attempt to understand the role of IL-32 in the pathogenesis of psoriasis, this is the first study to determine the role of IL-32 in the peripheral blood of psoriatic patients. The data revealed a marked increase of IL-32 gene expression in the majority of tested psoriatic patients and, as compared to healthy humans, the raised IL-32 levels in psoriasis patients not only suggest that inflammation is increased in psoriasis, but also indicate a potential role of IL-32 in the pathogenesis of psoriasis. 

To validate our central hypothesis that IL-32 is involved in the pathogenesis of psoriasis, the gene expression of IL-32 isoforms IL-32α, IL-32β, IL-32γ, and IL-32δ was demonstrated. Our data show that all tested isoforms of IL-32 α, β, γ, and δ were overexpressed in the PBMCs of psoriatic patients as compared with those of healthy humans. The data also pointed out that IL-32α was more significantly expressed as compared with other isoforms of IL-32, indicating that the major proinflammatory activity of IL-32 was derived from IL-32α in psoriasis. These findings are fully supported by other published reports, showing that IL-32α is the most abundant cDNA clone and the major proinflammatory activity of IL-32 was derived from IL-32α as it is involved in the activation of potent proinflammatory signaling events including p38-MAPK, ERK, JAK-STAT, and NF-κB in various cell types [13,23,24]. After IL-32α, the second most active isoform of IL-32 was found to be IL-32γ, although IL-32β and IL-32δ were also biologically active in psoriasis. Our results are somewhat consistent with previous findings on the identification of the active isoforms of IL-32 [44]. The increased levels of IL-32α observed in psoriatic patients in the present study, together with other isoforms of IL-32, provide further support for the involvement of IL-32 in psoriasis. Therefore, it is clear that IL-32 and its isoforms are implicated in contributing to the pathogenesis of psoriasis, which may be targeted to offer new therapeutic options for psoriasis. However, this study has a few limitations. The most obvious limitation of the study is the sample size; it would be better to include more patients in the study to rule out any inadequacy. In addition, patient selection criteria should be improved with more clinical diagnosis and the inclusion of more recently discovered IL-32 isoforms to further strengthen our findings. In this regard, further studies are recommended to comprehensively explore the therapeutic potential of IL-32 and its isoforms in psoriasis.

In conclusion, our results clearly show a significant increase of IL-32 levels in patients with chronic plaque psoriasis, suggesting that IL-32 is involved in the induction of inflammation in psoriasis. More importantly, the results of this study, for the first time, provide evidence of a strong association of IL-32 levels with the peripheral blood of psoriasis patients. To provide further support to these data, the study also records the mRNA levels of all major IL-32 isoforms (IL-32α, IL-32β, IL-32γ, and IL-32δ) in the peripheral blood of psoriasis patients. Among all IL-32 isoforms, IL-32α was most active, although all other isoforms were also active at the gene expression level. Longitudinal studies in psoriasis are necessary to further establish the role of IL-32 or its isoforms as contributing pathogenic mechanisms in psoriasis, and to assess their usefulness in evaluating the progression and severity of the disease, as well as in developing an effective target therapy for psoriasis.

## Figures and Tables

**Table 1:** Details of primers used in polymerase chain reaction.

Gene Name	Accession Number	Sense	Anti-Sense
IL-32	NM_004221	5′TCGCGGAGGTGGGTTTC3′	5′AAAACGGACTAATACGGCAACAG-3′
IL-32 α	NM_001012633.1	5′GCTGGAGGACGACTTCAAAGA3′	5′GGGCTCCGTAGGACTTGTCA3′
IL-32 β	NM_001012631.1	5′CAGTGGAGCTGGGTCATCTCA3′	5′GGGCCTTCAGCTTCTTCATGTCATCA3′
IL-32 γ	NM_001012635.1	5′AGGCCCGAATGGTAATGCT3′	5′CCACAGTGTCCTCAGTGTCACA3′
IL-32 δ	NM_001012636.1	5′TCTGTCTCTCTCGGGTCCTCTCT3′	5′TGTCTCCAGGTAGCCCTCTTTG3′
GAPDH	NM_002046	5′TCGACAGTCAGCCGCATCTTCTTT3′	5′ACCAAATCCGTTGACTCCGACCTT3′
ACTB	NM_001101	5′-AGAGCTACGAGCTGCCTGAC-3′	5′-AGCACTGTGTTGGCGTACAG-3′
